# Medical and Philosophical Intelligence

**Published:** 1812-01

**Authors:** 


### MEDICAL AND PHILOSOPHICAL INTELLIGENCE.

THE ROYAL SOCIETY met on the 7th of November, after the long vacation. At the meeting on the 21st, an Introduction to an experimental Inquiry into the nature, formation, and constituent parts, of the Blood, by Mr. Brande, was read. After noticing the difficulties and inaccuracies of all the methods of analysing animal matter, he examined the process adopted by Fourcroy and Vauquelin, and pointed out their errors, *and particularly the opinion of the existence of iron in the blood, which he asserts wholly to be unfounded.* He also took a review of the process by which the living animal converts its food into chyle, and thence into blood, investigated the nature of serum, and analysed that fluid with minute and scientific accuracy.

---

*Account of a Vegetable Wax from Brazil, by W. T. Brande, esq. F.R.S.*—The vegetable wax, described in this paper, was given to the president of the Royal Society by Lord Grenville, with a wish, on the part of his lordship, that its properties should be investigated, in the hope that it might prove a substitute for bees'-wax, and constitute a new article of commerce between the Brazils and this country. It is said to be the production of a tree of slow growth, called by the natives *Carnauba,* which produces a gum used as food for man, and another substance employed for fattening poultry. If this article can be procured in abundance, it may become a valuable addition to the comforts of mankind, by reducing the price and improving the quality of candles, flambeaux, &c. In the state in which it was sent to Lord Grenville, it resembles much that described by Humboldt as the produce of the *ceroxylon audicola,* but it is not likely to be the same, as Humboldt's wax is collected from a stately palm, which grows on high mountains. The Brazilian plant is described as a slow growing tree, but not as a large one. By the analysis of Vauquelin, the ceroxylon consists of two thirds resin, and one third wax; but the Brazilian article is entirely wax, and affords not the smallest trace of resin.

The wax, in its rough state, is in the form of a coarse grey powder, soft to the touch, and mixed with various impurities, which, when separated by a sieve, amount to about 40 per cent.

It has an agreeable odor somewhat resembling new hay, but scarcely any taste.

At 206° of Fahr. it enters into perfect fusion, and may then be further purified by passing it through fine linen. It acquires a dirty green color, and its peculiar smell becomes more evident; when cold is moderately hard and brittle; specific gravity 980.

Water exerts no action on the wax, unless boiled with it for some hours.

Alcohol dissolves no portion of the wax, unless heat be applied.

Two fluid ounces of boiling alcohol, specific gravity 826, dissolve about ten grains of the wax, of which eight are deposited as the solution cools; the remaining two may be afterwards precipitated by the addition of water.

Sulphuric ether, specific gravity 7563, dissolves a very minute portion of the wax, temperature 60°.

Two fluid ounces of boiling sulphuric ether dissolve 30 grains of the wax, of which 26 are deposited by cooling; the remaining four may be obtained by allowing the ether to evaporate.

The fixed oils very readily dissolve the wax at 212°, and form with it compounds of an intermediate consistence, very analogous to those obtained with common bees'-wax.

Some combinations of the vegetable wax with olive oil were perfectly soluble in ether, and sparingly soluble in boiling alcohol.

One hundred grains of the wax were boiled for half an hour in a solution of caustic potash, specific gravity 1090. The solution acquired a pale rose color, but appeared to exert no further action on the wax, which, after having been washed with warm water, retained its fusibility and other properties. No combination similar to a soap was produced.

The effects produced by boiling the wax in solutions of pure soda, and of the subcarbonates of soda, and of potash, were analogous to those of the caustic potash.

When the wax is boiled in nitric acid, specific gravity 1·45, there is some escape of nitrous gas, and the color of the wax is gradually changed to a deep yellow.

When the wax is removed from the acid, and washed with water, it is found to have become more brittle and hard, but still retains much of its peculiar odor. In this state it remains insoluble in the alkalies; but they now change its color to a very bright brown, which is destroyed by dilute muriatic acid, and the original color restored. Neither the fusibility nor the inflammability of the wax are impaired by this process.

Nitric acid, diluted with eight parts water, produces the same change in the color of the wax as the concentrated acid.

Mr. Brande was not successful in his attempt to bleach the wax in its original state; he found, by exposing it spread upon a glass to the action of light, it became in the course of three weeks of a pale straw color; on the surface nearly white. The same change was produced by steeping the wax in thin plates, in an aqueous solution of oxymuriatic gas, but did not render it perfectly white. Muriatic acid has little action upon the wax; when boiled upon it some hours, it destroys much of its color.

Sulphuric acid changes the color to a pale brown; and, when water is added, the wax becomes of a deep rose color: the inflammability and the fusibility are slightly impaired by this process.

When heat is applied, the wax is decomposed with the usual phenomena; sulphureous acid is developed, and charcoal deposited.

Acetic acid has very little action on the wax when cold: when boiled in this acid, a minute portion is dissolved, and again deposited as the solution cools: by long continued boiling in it, the wax is rendered nearly white; but then, if washed with water and fused, it resumes its former color. When fused in oxymuriatic gas, it is rapidly rapidly decomposed, and, parting with hydrogen and oxygen, muriatic acid and water are formed, and charcoal deposited. The results of the destructive distillation of the vegetable wax are very analogous to those of bees'-wax.

An acid liquor, mixed with a volatile oil, are the first products; these are succeeded by a large proportion of butyraceous oil, and a very small quantity of charcoal, affording traces of lime remains in the retort. During the process, a little carburetted hydrogen gas is given off.—Mr. Brande does not give the relative proportions of the different products, as they will vary according to the rapidity with which the distillation is conducted.

From the preceding experiments it appears, that, although the vegetable wax possesses the characteristic properties of bees'-wax, it differs from that substance in many of its chemical habitudes. It also differs from the other varieties of wax; namely, the wax of the *myrica cerifera*, of lac, and of white lac.

---

*Cure for the Fistula in Ano.*—Dr. Brown, of Islington, has stated in a reputable Magazine*, that a decoction of the blossoms of the *prunus silvestris*, have cured this disease. He relates three instances of its useful effects. A foreman of a brush-maker at Bristol, a person residing in Chiswell-street, and a smith at Islington, have, he says, been recently cured. The cure of the smith, as seeming most determined, we shall insert in his own words. This person, "with little hope of evading an operation, procured at a medical herb-shop in Covent-garden, some of the blossoms of the sloe-tree; an ounce of these were put into a coffee-pot of the ordinary size, to which he added three half-pints of water; it stood near the fire, simmering for some time, then was slightly boiled; the strained liquor, which looks like brandy, was taken two or three times a-day (the dose was four table-spoonfuls); and twice a-day he applied to the part affected, a bread and milk poultice, softened with ointment of marshmallows; and he is now well." Dr. Brown has heard that a person in the city has been cured of a very painful hæmorrhoidal complaint, solely by the use of the above decoction.

If we are slow to admit specific properties, attributed to substances on slight grounds, we think there is a probability that the petals of the *prunus spinosa* may be occasionally useful in diseases of the lower part of the intestinal canal. An infusion of a handful of these flowers, is said, by a late respectable physician and intelligent botanist, *to be a safe and easy purge.*

---

*The Art of Preserving all Kinds of Animal and Vegetable Substances for several Years.*—M. Appert has lately published, by order of the French minister of the interior, a work on the above subject, and which has been translated into English. M. Appert professes to have made considerable improvements in the art of preserving alimentary substances. The method proceeds on the principle that any substance, either animal or vegetable, may be preserved with-

* Month. Mag. Decem. 1811, p. 422.

out alteration for an indefinite length of time, without any addition, merely by excluding the air, and afterwards by subjecting the substance to the heat of boiling water. In this way are preserved different kinds of animal food, and fruits and vegetables of all descriptions; the first without salt or any kind of condiment, the latter without sugar.

*Brazil Salt.*—It was intended the public should believe that this salt was imported from the Brazils. Nearly twenty years ago it was discovered that this secret medicine was the *Rochelle salt*, mixed with a small proportion of brown sugar, and in a state of coarse powder instead of its beautiful crystals.

A young man, named John M'Isaac, of Corphine, in Kintyre, Scotland, made oath, on examination, at Campbeltown, before the sheriff-substitute of Kintyre, that he saw on the afternoon of the 13th of October last, on a black rock on the sea coast, an animal, of the particulars of which he gives a long and curious detail, answering in general to the description commonly given of the supposed amphibious animal, called the Mermaid. He states, that the upper part of it was white, and of the shape of a human body; the other half, towards the tail, of a brindled or reddish grey color, apparently covered with scales; but the extremity of the tail itself was of a greenish red shining color; that the head was covered with long hair; at times it would put back the hair on both sides of its head, it would also spread its tail like a fan; and while so extended, the tail continued in tremulous motion, and, when drawn together again it remained motionless, and appeared to the deponent to be about twelve or fourteen inches broad; that the hair was long and light-brown; that the animal was between four and five feet long; that it had a head, hair, arms, and body, down to the middle, like a human being; that the arms were short in proportion to the body, which appeared to be about the thickness of that of a young lad, and tapering gradually to the point of the tail; than when stroking its head, as above-mentioned, the fingers were kept close together, so that he cannot say whether they were webbed or not; that he saw it for near two hours, the rock on which it lay being dry; that after the sea had so far retired, as to leave the rock dry to the height of five feet above the water, it tumbled clumsily into the sea; a minute after he observed the animal above water, and then he saw every feature of its face, having all the appearance of a human being, with very hollow eyes. The cheeks were of the same color with the rest of the face; the neck seemed short; and it was constantly, with both hands, stroking and washing its breast, which was half immersed in the water. He therefore cannot say whether its bosom was formed like a woman's or not. He saw no other fins or feet upon it, but as described. It continued above water for a few minutes, and then disappeared. He was informed that some boys in a neighbouring farm saw a similar creature in the sea, close to the shore, on the same day. The minister of Campbeltown, and the chamberlain of Mull, attest his his examination, and declare they know no reason why his veracity should be questioned.

---

Among the improvements which are constantly proceeding in this great metropolis, conducing to its salubrity as well as beauty, it has long been a matter of surprise, that accommodation for bathing, on a large and liberal scale, has not been afforded. Though the town is actually washed by a noble river, and the suburbs are intersected by canals, the inhabitants may not plunge into the refreshing stream, however oppressed with heat. We have therefore great satisfaction in stating that a plan has been formed, and will be brought before parliament early in the ensuing session, for establishing public sea-water baths on an extensive scale. To effect this, it is proposed to lay iron pipes (forming a main) from the coast of Essex to Copenhagen fields, or some such elevated and eligible situation; where a reservoir, covering several acres of land, will be formed, into which, by means of a powerful steam engine, a constant supply of sea-water will be continually kept flowing. From this grand reservoir, the numerous baths would be supplied with a running stream, conveyed by fountains of various descriptions.

It is also proposed to connect with this establishment every species of warm, cold, shower, and vapor, baths; and the spacious grounds, which will include a botanic garden, are to be laid out in tasteful walks, shrubberies, and lawns, forming splendid promenades, conducive equally to the purposes of health and amusement. A laboratory for preparing artificial mineral waters, and the gases used in pneumatic medicine, and apparatus for inhaling them, will also be provided. Under the patronage of the Prince Regent, all the male branches of the royal family, supported by several of the nobility, and under the direction of a most respectable committee, little doubt can be entertained that the project, combining so many public advantages, will be carried into effect; and, as it proceeds towards maturity, we shall most probably have occasion to refer to it on a future occasion.

---

Among the new patents granted since the middle of September, the following have a connection with the practice of medicine:—To Johannes Ambrosius Maas, of Hammersmith, in Middlesex, for an improvement in making vinegar.—To William Strahan, of Poole-cottage, in the county of Chester, chemist, for a new method of making salt.—To John Miers, of the precinct of the Savoy, Middlesex, jeweller, for a new method of accelerating the evaporation of liquid or fluid bodies, destroying the noxious and offensive effluvia arising from spent soap lyes, or other liquid, fluid, or solid, substances, and generated by an increased degree of heat without additional fuel.—To John Lowndes, of Hollen-street, Soho, in Middlesex, modeller, for an improved method of heating baths.

---

Of the late discoveries of the Russian travellers, that of an island in the icy ocean, by *Syrawatskoi*, a merchant, deserves particular attention. Hedemstrom, the Russian naturalist, who has recently examined this island, which has received the appellation of New Siberia, found three claws, of some bird, a yard in length; and the roving Jakute related, that they had sometimes found feathers, the barrels of which were capable of admitting a man's clenched fist.

---

It has been publicly announced in the St. Vincent's Gazette, that Dr. Anderson has succeeded this year in preserving a quantity of clove-seed, fit for the propagation of that valuable spice; and that persons inclined to cultivate it in that island, might be furnished by him with seed.

---

Mr. Haworth, of Little Chelsea, has it in contemplation to publish, in an 8vo. volume, a Description of the succulent Plants that are at present, or have recently been, cultivated in the gardens in the neighbourhood of London; comprising upwards of seven hundred species, nearly the whole of which are at present in his possession.

---

The first volume of an American Ornithology is publishing at Philadelphia. This work will contain the natural history of the birds of the United States, comprehending those which reside, and those which migrate from other regions. Among those described, or intended to be described in this work, are many both land and water birds not hitherto noticed.

---

The delay which has taken place in the publication of Dr. Willan's valuable work on Cutaneous Diseases, has not been occasioned, we understand, by his recent indisposition, nor by his voyage to Madeira; but is owing principally to the negligence of the publishers. Part of his MS. has been ready for the press a considerable time.

---

Dr. Bateman intends to publish a practical Synopsis of the whole of Dr. Willan's classification, for the purpose of exhibiting a concise view of the characteristic symptoms of each disease, and of the methods of treatment, divested in a great measure of the literary and historical discussions.

---

On the 1st of January will be published, a Dissertation on the Bite of a Rabid Animal, by James Gillman, surgeon, Highgate; being the substance of an Essay which received a prize from the Royal College of Surgeons in London.

---

Dr. Sutton has in the press, Observations on the injurious Effects of Mercury in various Diseases.

---

Mr. Davy has in the press a first volume of Elements of the Philosophy of Chemistry.

---

Dr. Clough will commence his next course of Lectures on the Science and Practice of Midwifery, including the Diseases of Women and Infants, as connected therewith, on Monday morning the 13th of January, 1812, at half-past ten; and on the evening of the same day day at seven; and every Monday, Wednesday, and Friday, at the same hour. These lectures will be elucidated by appropriate specimens from a very valuable collection, and the student's views forwarded, by being amply provided with cases for practical improvement, when properly qualified to attend them. An Obstetrical Converzatione will be held once a fortnight, which none but pupils can attend.

---

Dr. Adams's spring course of Lectures on the Institutes and Practice of Medicine, will be commenced at his house, No. 17, Hatton Garden, about the beginning of January, 1812.

---

Mr. Armiger, surgeon extraordinary to his R. H. the Duke of Kent, and Surgeon to the Eastern Dispensatory, will commence his spring course of Lectures on Anatomy, Physiology, and Surgery, on Monday, January the 20th, 1812, at the Theatre of Anatomy, Bartlett's-court, Holborn.

---

Dr. Clutterbuck will begin his spring course of Lectures on the Theory and Practice of Physic, Materia Medica, and Chemistry, on Monday the 20th of January, at ten o'clock in the morning, at his house, No. 1, in the Crescent, New Bridge-street. The different lectures are given on alternate days: viz. Practice of Physic on Mondays, Wednesdays, and Fridays; Materia Medica and Chemistry on Tuesdays, Thursdays, and Saturdays, at the same hour.

---

Dr. Clarke and Mr. Clarke will commence their spring course of Lectures on Midwifery and the Diseases of Women and Children, on Monday, January the 27th. The lectures are read every day at the house of Mr. Clarke, No. 10, Upper John-street, Golden-square, from a quarter past ten o'clock in the morning, till a quarter past eleven, for the convenience of students attending the hospitals.

---

Dr. Ramsbotham will commence his next course of Lectures on the Science and Practice of Midwifery, and the Diseases of Women and Children, on Thursday, January 9th, 1812, at his house, No. 9, Old Jewry, at half after five in the evening.

---

Lectures on the Theory and Practice of Midwifery, illustrated by a variety of Cases, at the Westminster Lying-in Institution for attending the Wives of Soldiers and Sailors, &c. Queen-square, Westminster. Under the conduct of J. Hopkins, surgeon to his R. H. the Duke of Kent, and W. Pearson, surgeon of the Royal College.—The resident pupils are to attend cases in rotation, and it is engaged that each of them do deliver one patient every nine days, or oftener; and that the attending pupils do deliver one every twenty-eight days. The number of gentlemen to engage, at the commencement of a course, is governed by that of the patients then appearing on the books.

---

*Russell Institution.*—A course of Twelve Lectures on Electricity will be commenced on Thursday, the 9th of January, 1812, by Mr. George Singer, at eight o'clock in the evening.—The intimate connection connection of electrical science with other important branches of natural knowledge, and the want of a popular exposition of its principles, have induced Mr. Singer to select this subject, as one the best calculated to excite interest, and promote useful inquiry. The various phenomena of electrical action will be systematically arranged, and illustrated by numerous experiments on a most powerful and extensive apparatus. The natural agencies of electricity will be investigated; and the phenomena and effects of thunder, lightning, meteors, &c. explained and demonstrated. In considering the chemical effects of electricity, the brilliant experiments on the productions of the metallic oxyds, (as effected by the large apparatus at Haerlem,) will be repeated, and some curious results of similar experiments exhibited. The voltaic battery will also be considered, and illustrated by original experiments, and by reference to the analysis of Mr. De Luc.

---

*Theatre of Anatomy, Blenheim-street, Great Marlborough-street.*—The spring course of Lectures on Anatomy, Physiology, and Surgery, will be commenced on Monday, the 20th of January, at two o'clock, by Mr. Brookes. Anatomical Converzationes will be held weekly, when the different subjects treated of will be discussed familiarly, and the students' views forwarded: to these none but pupils can be admitted. Spacious apartments, thoroughly ventilated, and replete with every convenience, are open all the morning, for the purposes of dissecting and injecting, where Mr. Brookes attends to direct the students, and demonstrate the various parts as they appear on dissection. The inconveniences usually attending anatomical investigations, are counteracted by an antiseptic process. Pupils may be accommodated in the house. Gentlemen established in practice, desirous of renewing their anatomical knowledge, may be accommodated with an apartment to dissect in privately.

---

*Middlesex Hospital.*—The following Lectures will re-commence at this hospital early in January:—On the Practice of Physic, and Diseases which occur in the Hospital, by Dr. Satterley. On the Theory and Practice of Midwifery, by Dr. Merriman. Mr. Cartwright will undertake such occasional Demonstrations of Morbid Anatomy, as may be required for the illustration of the respective cases.

---

*London Hospital.*—Dr. Buxton's spring course of Lectures on the Practice of Medicine, will be commenced about the 20th of January, 1812.

---

*Account of a new Volcanic Island.*—His majesty's sloop Sabrina arrived in the Tagus lately from a cruize off the Western Isles, or Azores, and brought the following account:—On the 16th of June they observed two columns of white smoke arising from the sea, off the west end of the island of St. Michael's, which for some time they supposed to be an engagement, and made all sail towards it; but were prevented, by the wind dying away. The smoke continued to ascend with sometimes large flames of fire, and they then concluded that it was a volcano. Next day they were close in with the island of St. Michael's, and found the volcano situated about two miles west of that island, and still raging in the most awful manner. They learnt from the British consul at St. Michael's, that smoke was first observed arising from that place on the 14th of June; previous to which there had been several very severe shocks of an earthquake felt at St. Michael's, so that the destruction of the whole island was much feared; but they ceased as soon as the volcano broke out. On the 18th the Sabrina went as near the volcano as she could with safety, and found it still raging with unabated violence, throwing up from under the water large stones, cinders, ashes, &c. accompanied with several severe shocks. About noon on the same day they observed the mouth of the crater just showing itself above the surface of the sea, where there were formerly 40 fathoms, or 240 feet, of water. They christened it 'Sabrina Island.' At three p. m. same day, it was about 30 feet above the surface of the water, and about a furlong in length. On the 19th they were within five or six miles of the volcano, and found it about 50 feet in height, and two thirds of a mile in length; still raging as before, and throwing up large quantities of stones, some of which fell a mile distant from the volcano. The smoke drew up several water-spouts, which, spreading in the air, fell in a heavy rain, accompanied with vast quantities of fine black sand, which completely covered the Sabrina's decks at the distance of three or four miles from the volcano. On the 20th they went on a cruize, leaving the volcano about 150 feet high, and a mile in length, still raging as formerly, and continuing to increase in size. On the 4th of July they again visited the volcano, and found it perfectly quiet. They went on shore on Sabrina Island (as it is now called), and found it very steep; its height not less than from 200 to 300 feet. It was with difficulty they were able to reach the top of the island; which they at last effected, in a quarter where there was a gentle declivity; but the ground, or rather the ashes, composed of sulphureous matter, dross of iron, &c. was so very hot for their feet, that they were obliged soon to return. They, however, took possession of the island in the name of his Britannic Majesty, and left an English union-jack flying on it. The circumference is now from two to three miles. In the middle is a large basin full of boiling-hot water, from which a stream runs into the sea; and, at the distance of 50 yards from the island, the water, although 30 fathoms deep, is too hot for one to hold his hand in. In short, the whole island is but a crater; the cliff on the outside appearing as walls, as steep within as they are without. The basin of boiling water is the mouth from which the smoke, &c. issued. When the Sabrina left it, several parts of the cliff continued to smoke a little; and it was their opinion that it would soon break out again.—I presume you are informed of this strange phænomenon before now: however, as I had the foregoing account from a young gentleman belonging to the Sabrina, who was an eye-witness of what is related, I conceived it likely to contain some particulars of which you have hitherto been uninformed."

 The

The following circumstantial account of three Meteoric Stones, which fell near Orleans, is translated from M. de la Metherie's Journal:—"On the 25th of November, 1810, at half past one in the afternoon, three atmospheric stones fell perpendicularly at Charsonville, in the department of Loiret. Their fall was accompanied with a succession of thunder-claps which preceded them and lasted some minutes. The noise of these explosions, in number three or four, followed by the roll produced by the echo, was heard as distinctly at Orleans as at the place where the stones fell. It is even said the noise was as loud at Montargis, Salbri, Vierzon, and Blois, as in each of these places it was the cause of some alarm, and was attributed to the explosion of a powder-mill. It is concluded, that, in consequence of the great distances in the circle in which the noise was heard, the explosion took place at a height in the atmosphere almost incalculable. The stones were found within an extent of half a league of each other; and their fall, in a perpendicular direction, was without any apparent light or globe of fire attending them. One of the stones, which fell at Mortelle, it seems had not been found. Another fell at Villeroi, and the third at Moulinbrûlé. One of them weighed twenty pounds, and made a hole in the ground, in a vertical direction, just big enough to bury itself, at the same time that it threw up the earth eight or ten feet high. This stone was taken out about half an hour afterwards, being still hot enough to be held in the hand with some difficulty. It diffused a strong scent like that of gunpowder, which it retained till it was perfectly cold. The second stone made a hole similar to the other in a vertical direction, and, being found eighteen hours after its fall, was quite cold. These stones were irregular in their shape, and their angles in general obtuse; they contained rather more globules of iron than those that fell at l'Aigle, in Normandy; these globules are also rather larger, and the colour of the stone when first broken is somewhat clearer; it may be speedily oxyded, and is sufficiently dense and heavy to write upon glass. It is broken with difficulty, and comes to pieces very irregularly, and is very fine in the grain. Its exterior is about a quarter of a line in thickness, and its colour of a darkish grey. These stones are also traversed by some irregular black lines, strongly marked, from half a line to two lines thick, and which traverse them in a manner similar to the veins of certain rocks.—Does not this fact seem to indicate that they existed prior to their fall, that they have been produced in the same manner as rocks, and were not formed in the atmosphere?

